# Tumor-derived extracellular vesicles mediate cell-specific uptake and facilitate enhanced doxorubicin delivery in breast cancer

**DOI:** 10.3389/fphar.2025.1744895

**Published:** 2026-01-12

**Authors:** Dhananjay B. Alagundagi, Mahima Rachel Thomas, Vinay C. Sangamesh, Vinay Kumar J Rajendra, Vijith V. Shetty, Shama Prasada Kabekkodu, Praveenkumar Shetty, Prakash Patil

**Affiliations:** 1 Central Research Laboratory, K S Hegde Medical Academy, NITTE (Deemed to be University), Mangaluru, Karnataka, India; 2 NITTE University Centre for Science Education and Research, NITTE (Deemed to be University), Mangaluru, Karnataka, India; 3 Department of Oncology, Justice K S Hegde Charitable Hospital, K S Hegde Medical Academy, NITTE (Deemed to be University), Mangaluru, Karnataka, India; 4 Department of Cell and Molecular Biology, Manipal School of Life Sciences, Manipal Academy of Higher Education, Manipal, Karnataka, India; 5 Department of Biochemistry, K S Hegde Medical Academy, NITTE (Deemed to be University), Mangaluru, Karnataka, India

**Keywords:** breast cancer, cellular uptake, doxorubicin, drug delivery, extracellular vesicles, nanocarriers

## Abstract

**Background:**

Chemotherapy suppresses tumor growth and metastasis, but its efficacy is limited by non-specificity, systemic toxicity, poor accumulation and side effects. Extracellular vesicles (EVs) derived from cells have recently been tested for carrying drugs due to their biocompatibility, stability, and ability to cross biological barriers. We aimed to investigate the potential of drug incorporation to breast cancer (BC) cells-derived EVs and their targeted cell-specific delivery.

**Methods:**

EVs were isolated from MDA-MB-231 and MCF7 cells and characterized by nanoparticle tracking analysis and scanning electron microscopy. The cellular uptake of EVs assessed by PKH67 labelling and fluorescent microscopy. Doxorubicin (dox) was incorporated into EVs by sonication, entrapment was confirmed by high-performance liquid chromatography (HPLC). Further, BC cells cytotoxicity, apoptosis, and wound healing was determined for therapeutic efficacy of dox-loaded EVs compared to free dox.

**Results:**

EVs had average size of 126.6 ± 58.6 nm (MDA-MB-231) and 163.3 ± 25.7 nm (MCF7), with spherical morphology. EVs exhibited significantly higher autologous uptake compared to allogenic, heterologous or non-cancerous uptake, confirming parent cell-type specificity. Dox entrapment was 22.84% and 29.87%. Furthermore, dox-EVs reduced parent cell viability to 46.1% (MDA-MB-231) and 35.3% (MCF7) compared to free dox treatment (63.4% and 62.1%). Additionally, dox-EVs suppressed wound healing and enhanced apoptosis more effectively than free Dox, while EVs alone promoted cell proliferation.

**Conclusion:**

Overall, EVs uptake is cell-specific, and drug incorporation enhanced targeted cell cytotoxicity, highlighting their potential as personalized carriers for precision chemotherapy. However, validation of these results through mechanistic and *in vivo* studies are warranted to extrapolate for therapeutic potential.

## 1 Introduction

Breast cancer (BC) is the most prevalent cancer among women worldwide and is associated with significant cancer-related morbidity and mortality. Its incidence continues to rise at an annual rate of 1%–5%, especially among the Asian and African populations. The recent global estimates indicate approximately 2.3 million new cases and over 680,000 deaths per year, underscoring the increasing health burden (Sung et al., 2021; Kim et al., 2025). Despite advances in multimodal management such as surgery, radiotherapy, chemotherapy, endocrine therapy, targeted drugs, and immunotherapies, clinical outcomes are often constrained by late-stage presentation, non-specific drug distribution systemic toxicity, drug resistance, and tumor recurrence (Sun et al., 2017; Schick et al., 2021; Wang and Wu, 2023). These challenges underscore the urgent need for more precise, biocompatible, and easily accessible therapeutic strategies that can enhance efficacy while reducing off-target effects.

Nanocarrier-based drug delivery systems, such as liposomes and polymeric nanoparticles, have demonstrated enhanced drug pharmacokinetics and bioavailability (Din et al., 2017; Cong et al., 2024). These systems can encapsulate various drugs, enhance drug stability, and facilitate controlled release (Hossen et al., 2019; Alshawwa et al., 2022). However, their clinical translation has been constrained by rapid clearance, immune activation, and poor targeting specificity. In this regard, cell-derived extracellular vesicles (EVs) have emerged as next-generation natural nanocarriers with the potential to overcome the challenges posed by synthetic systems. EVs are membrane-bound vesicles, secreted by almost all cell types, and play a crucial role in intercellular communication, especially within the tumor microenvironment (Yáñez-Mó et al., 2015). They are broadly classified as exosomes (30–150 nm), derived from the fusion of multivesicular bodies with the plasma membrane; microvesicles (MVs) (100–1,000 nm), formed by outwards budding from the plasma membrane; and apoptotic bodies (>1,000 nm), generated during programmed cell death (Doyle and Wang, 2019; Lopez et al., 2023). EVs contain diverse cargos, including proteins, lipids, and nucleic acids, and deliver them to recipient cells through receptor-mediated interactions, thereby influencing cellular signaling, behavior and proliferation (Zemanek et al., 2025). In this way, EVs stand out as an ideal candidate for transporting therapeutic agents to target cells due to their origin, inherent biocompatibility and natural ability to traverse biological barriers. Unlike the synthetic nanocarriers, cell-derived EVs exhibit low immunogenicity, prolonged circulation, and cell-specific tropism. These unique features facilitate cell-specific targeting, enhancing drug accumulation at the tumor site while minimizing systemic toxicity (Frolova and Li, 2022; Wang et al., 2023).

Doxorubicin (Dox) is an anthracycline chemotherapeutic agent that induces cell cycle arrest and apoptosis by DNA intercalation and inhibition of topoisomerase-II (Wang et al., 2019; Ma and Zhang, 2020). It is widely used for the treatment of various cancers, including BC. Though implicated across different stages of BC including metastatic, aggressive triple-negative breast cancer (TNBC), the clinical application of dox is restricted by non-specific toxicity, resulting in side effects such as cardiotoxicity (Kong et al., 2022) and myelosuppression (Bhinge et al., 2012), and multidrug resistance (Christowitz et al., 2019) which further limits the long-term use and reduces therapeutic efficacy (Xia and King, 2025). In such cases, EVs can be encapsulated with dox (dox-EVs) and delivered to the target cell, improving intracellular bioavailability, minimizing systemic toxicity, and reducing off-target side effects. Additionally, cancer cell-derived EVs may exhibit selective uptake by their parental (autologous) cells, leveraging natural homing mechanisms mediated by membrane proteins and lipid signatures.

In this context, the present study investigated the potential of BC cell-derived EVs as cell-specific drug delivery vehicles. We hypothesized that these EVs would demonstrate preferential uptake by their parent cells (autologous) and that drug-loaded autologous EVs would enhance the cytotoxic effect in parent cells, compared to non-parent cells (allogenic). To test our hypothesis, we employed two widely used BC cell lines representing different molecular and invasive phenotypes: an aggressive and highly invasive TNBC cell line, MDA-MB-231 and the less invasive and hormone receptor-positive MCF7 cell line. Both models represent key biological contrasts within the BC spectrum. Our findings underscore the potential of EV-based therapeutics, offering improved specificity and efficacy while mitigating systemic toxicity, and paving the way for the development of more precise and patient-friendly cancer therapies.

## 2 Materials and methods

### 2.1 Cell culture

MDA-MB-231 and MCF7 cell lines were cultured as monolayers in Dulbecco’s modified Eagle’s medium supplemented with 10% fetal bovine serum. Additionally, 1,000 U/mL penicillin and 1,000 U/mL streptomycin were added to the medium to prevent bacterial contamination. The cells were maintained in a humidified incubator at 37 °C with 5% CO2 to ensure optimal growth conditions and physiological pH balance. The patient-derived human foreskin fibroblasts (HFFs) and oral squamous cell carcinoma (OSCC) cells were a kind gift from Dr. Ajaykumar Oli (SDM College of Medical Sciences and Hospital, Dharwad, Karnataka, India). Both cell lines were maintained under the same culture conditions and growth medium as mentioned above.

### 2.2 Isolation of extracellular vesicles

EVs from MDA-MB-231 and MCF7 cells were isolated as previously reported (Eswaran et al., 2025). Briefly, cells were cultured in five T75 cell culture flasks to 80% confluency and their growth medium were switched to a serum-free medium for 24 h. Then, conditioned medium was collected from the plates and subjected to differential centrifugation: 300 × g for 10 min, 3,000 × g for 20 min, and 10,000 × g for 30 min to remove floating cells and cell debris. Afterward, the conditioned medium was ultracentrifuged at 100,000 × g for 120 min using an Optima XE Beckman Coulter ultracentrifuge; the supernatant was discarded, and the EV pellet was washed with ice-cold phosphate-buffered saline (PBS), followed by one additional round of ultracentrifugation. Finally, the collected EV pellet was resuspended in PBS and stored at −80 °C until use (Théry et al., 2018).

### 2.3 Characterization of extracellular vesicles

Isolated EVs were characterized according to the Minimum Information for Studies of Extracellular Vesicles (MISEV) 2018 guidelines (Théry et al., 2018). The EV samples were vortexed to prevent clumping and diluted 1:200 with sterile PBS to achieve consistent particle concentrations before characterizing them for size and concentration using a NanoSight NS300 (Malvern Instruments, Malvern, United Kingdom). Six independent experiments were performed to identify and confirm the size and particle counts of the EVs. EV’s size distributions, mean size, particle count, and standard deviations (SD) were calculated using the software (NTA 3.4). The surface charge of the EVs was analyzed via a Horiba SZ-100V2 Zetasizer. For scanning electron microscopy (SEM) imaging, EVs were mounted on aluminum holder stubs using double-sticky carbon tape, coated with Au/Pd in a SPI-MODULETM high-resolution sputter coater and examined under a microscope (EVO LS 15, ZEISS, Germany).

### 2.4 PKH67 labelling of extracellular vesicles

EVs were labeled with a PKH67 Green Fluorescent Cell Linker Kit (MINI67, Sigma‒Aldrich, St. Louis, MO, United States of America). Briefly, 100 µL of the EVs were diluted to 500 µL with Diluent C, and the dye was added to achieve a final concentration of 3 µM. The solution was mixed by gentle pipetting for 30 s and incubated for 30 min at room temperature in the dark. After incubation, the mixture was centrifuged twice at 14,000 × g for 5 min each and then ultracentrifuged 100,000 × g for 60 min to remove the dye aggregates. The final pellet was resuspended in PBS and stored at −80 °C until further use.

### 2.5 Assessment of cellular uptake of extracellular vesicles

BC cell lines (1 × 10^5^) were seeded on coverslips kept in a six-well plate; once they reached 50% confluency, they were treated with 50 µL of labeled EVs, autologous and allogenic, for different time intervals (0, 3, 6 and 12 h). After each time point, the spent media was discarded, and the cells were washed with PBS before fixation with 3.7% paraformaldehyde for 10 min. Subsequently, the cells were washed and permeabilized with ice-cold methanol for 45 min at −20 °C. The methanol was then removed by washing the cells thrice with PBS, followed by staining with DAPI for 30 min. Finally, the cells were washed with PBS, mounted and observed via a fluorescence microscope (BX53-Olympus, Japan and D2500 Leica, Germany). Furthermore, the cellular uptake of BC cell-derived EVs was assessed in patient-derived HFFs and OSCC cells to examine their specificity towards non-cancerous and heterologous cancer cell types.

### 2.6 Preparation, characterization of doxorubicin-loaded extracellular vesicles (dox-EVs) and high-performance liquid chromatography

Dox was loaded in BC cell-derived EVs according to previous reports (Kim et al., 2016). Briefly, 150 µg of isolated EVs were mixed with 50 µL of 10 µM dox and subjected to six cycles of probe sonication at 20% amplitude, with 30 s on/off pulses and a 2-min cooling interval between each cycle to prevent heat-induced damage. Following sonication, the mixture was incubated at room temperature for 60 min to promote entrapment. After incubation, the samples were centrifuged three times (3,300 × g for 10 min at 4 °C) to remove unbound dox. The final pellet was dissolved in PBC and the presence of entrapped dox in EVs was then quantified by measuring the absorbance at 488 nm via a spectrophotometer (Spark multimode reader, Tecan, Switzerland). A standard curve of dox was prepared to determine the entrapment efficiency (EE), and the EE was calculated via the formula EE (%) = (OD of Dox in EV fraction/OD of free dox) X 100.

High-performance liquid chromatography (HPLC) analysis was performed using a Waters Alliance HPLC system (Waters Corporation, Milford, MA, United States of America) to evaluate dox loading. First, 250 µL of EV-dox was heated to 75 °C, and an equal volume of acetonitrile was added. The mixture was then vortexed, sonicated and centrifuged at 13,000 × g for 10 min. The supernatant was filtered, and 20 µL of the sample was injected and run onto a CUnited States column. The separation was performed using a mobile phase of acetonitrile: water (55:45) (v/v) at a flow rate of 1 mL/min at 30 °C. The absorbance was measured at 230 nm to monitor the elution of dox.

### 2.7 Cytotoxicity assay

MDA-MB-231 and MCF7 cells were treated with free dox, EVs alone or dox-EVs to assess the targeted cytotoxic potential of dox-loaded EVs. 5 × 10^3^ cells per well were seeded in 96-well plates. The cells were maintained in DMEM containing 1% serum for 12 h and then treated with dox (2 μM), EVs (150 μg/mL) or dox-EVs (150 μg/mL) for 48 h. Cell viability was then evaluated via the MTT assay (3-(4,5-dimethyl-2-thiazolyl)-2,5-diphenyl-2-H-tetrazolium bromide), and the absorbance was measured at 570 nm. For cytotoxicity and other functional assays, dox-EV concentrations were normalized to their actual dox content based on encapsulation efficiency.

### 2.8 Scratch wound healing assay

MDA-MB-231 and MCF7 cells were cultured in 6-well plates until 80% confluency, followed by serum starvation for 24 h. A linear scratch was made at the center of the cell monolayer via a sterile microtip. The cells were treated with DMEM containing 1% serum supplemented with dox, EVs or dox-EVs at the abovementioned concentrations. Wound closure and cell migration into the scratch area were monitored at designated time points via a CKX53 inverted microscope (Olympus, Japan) equipped with a digital camera.

### 2.9 Analysis of apoptosis by annexin V-FITC

The apoptotic cells were quantified using Dead Cell Apoptosis Kits with Annexin V for flow cytometry (Thermo Fisher Scientific) following the manufacturer’s protocol. Briefly, cells were seeded at a density of 0.3 × 10^6^ cells per well in six-well plates and incubated for 24 h. The cells were treated with EVs, dox-EVs, or dox for 48 h. After treatment, the cells were harvested and resuspended in 300 µL of 1X annexin-binding buffer. Staining was performed by adding 5 µL of Annexin V-FITC and 1 µL of propidium iodide (PI), followed by incubation at room temperature in the dark for 15 min. The apoptotic index was immediately analyzed via a BD Accuri™ C6 Plus flow cytometer (BD Biosciences, United States of America).

### 2.10 Statistical analysis

Statistical analysis was performed via unpaired t-test or one-way ANOVA using GraphPad Prism software. The values are expressed as the mean ± SD. The results with *p*-values <0.05 were considered statistically significant. All experiments were performed in duplicate and repeated three times independently.

## 3 Results

### 3.1 Cell-specific extracellular vesicles have different sizes, morphologies, and surface charge

**FIGURE 1:**
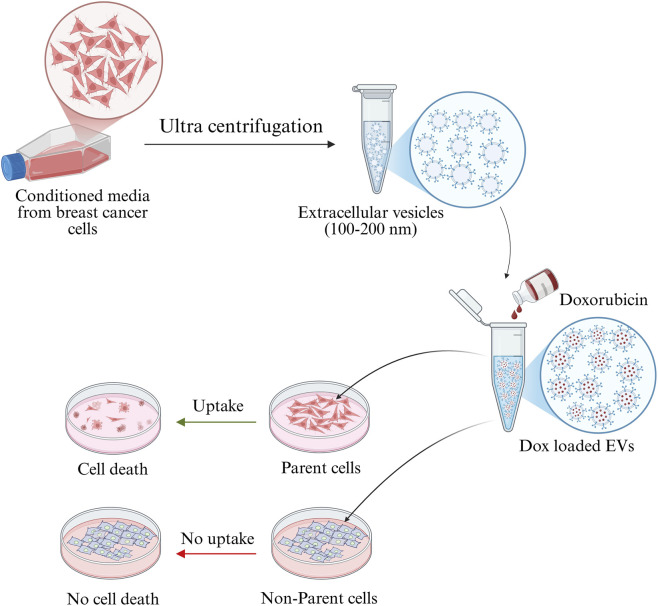
Graphical abstract showing that BC cancer cells-derived EVs internalized specifically by parent cell and deliver the drug thereby inducing the cell death.

**FIGURE 2:**
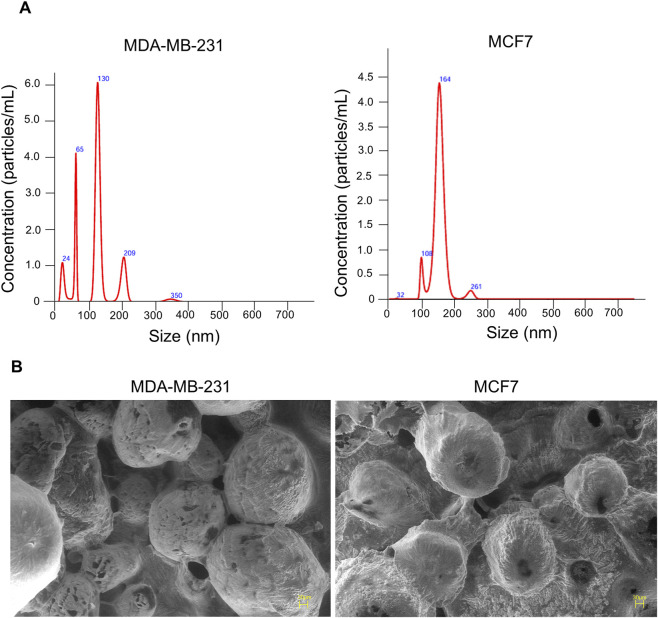
Characterization of EVs isolated from MDA-MB-231 and MCF7 cells. **(A)** EVs size distribution and particle count were analysed via NTA and surface charge measured via zeta potential. **(B)** Scanning electron microscopy (SEM) analysis of EVs reveals their ultrastructure, showing predominantly round or oval-shaped particles.

The schematic representation of the study was presented in Figure 1. EVs were isolated from MDA-MB-231 and MCF-7 cell lines and characterized for size, morphology, and surface charge. The nanoparticle tracking analysis revealed that MDA-MB-231-derived EVs had an average diameter of 126.6 ± 58.6 nm, while MCF-7-derived EVs were larger, with an average diameter of 163.3 ± 25.7 nm, suggesting cell line-specific differences in EV biogenesis. The polydispersity indices were 0.32 and 0.27 for MDA-MB-231 and MCF-7 EVs, respectively, indicating moderately heterogeneous populations. SEM analysis revealed spherical morphology of both EV types. Zeta potential measurements revealed the surface charges on EVs: −0.2 mV for MDA-MB-231-derived EVs and −2.5 mV for MCF-7-derived EVs (Figure 2). These results highlight that BC cell-derived EVs possess cell-specific physicochemical properties.

### 3.2 Breast cancer cell-derived extracellular vesicles exhibited a cell-specific uptake

**FIGURE 3:**
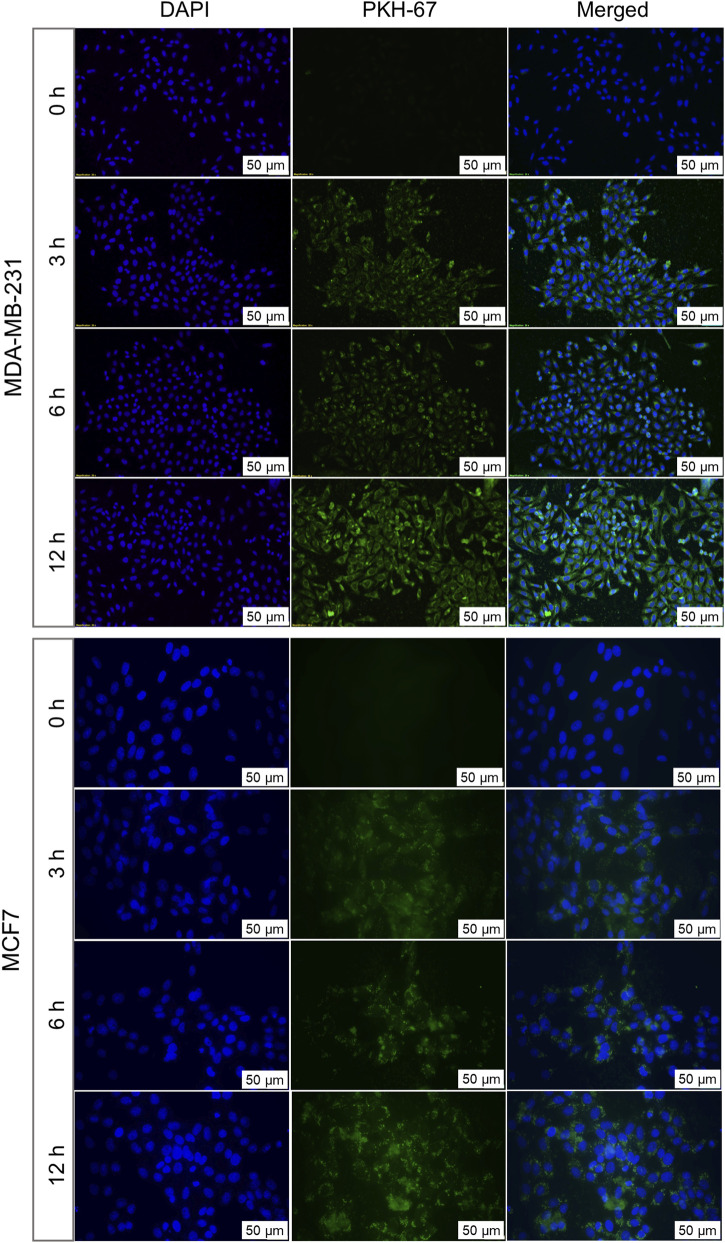
Autologous uptake of BC cell-derived EVs analysed by fluorescence microscopy. MDA-MB-231-derived EVs labelled with PKH67 were incubated with their parent cells for 3, 6, and 12 h MCF7-derived EVs labelled with PKH67 were similarly incubated with their parent cells. Fluorescent signals indicate time-dependent internalization of EVs by cells over time. Nuclei were counterstained with DAPI (blue). Representative images from three independent experiments are shown.

**FIGURE 4:**
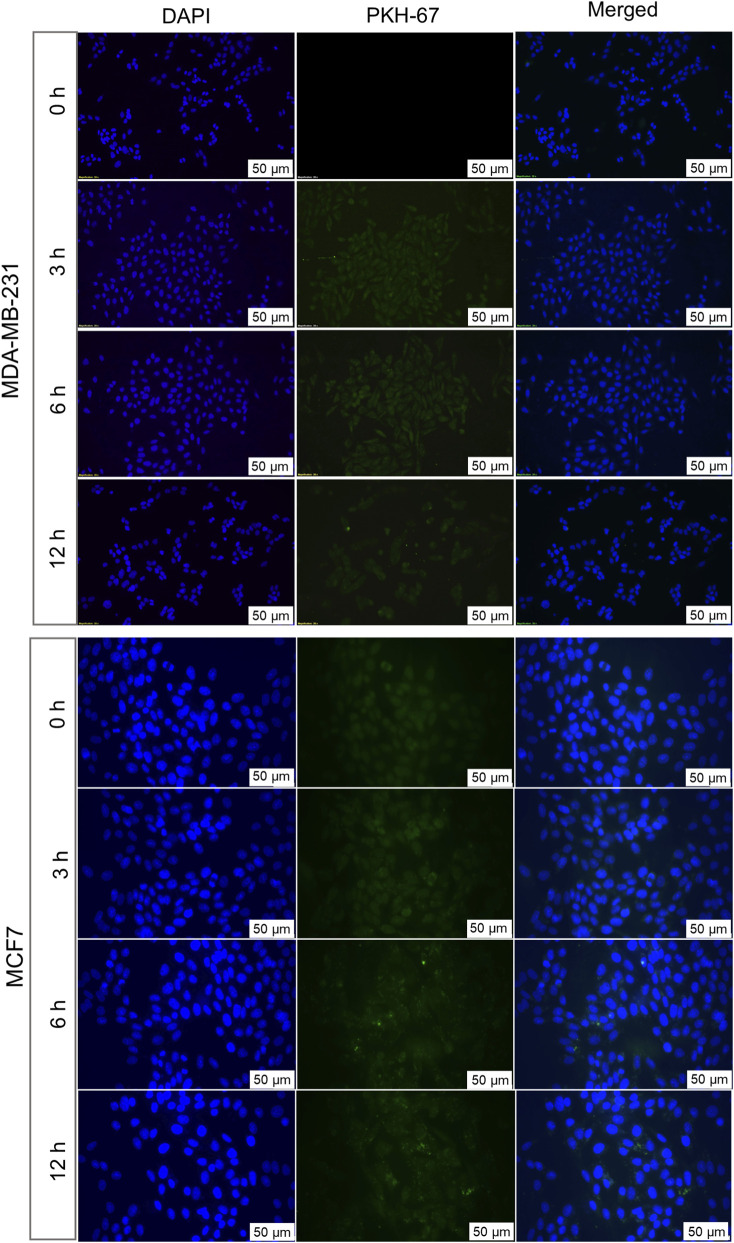
Allogenic uptakes of BC cell-derived EVs analysed by fluorescence microscopy. MCF7-derived EVs labelled with PKH67 were incubated with MDA-MB-231 cells for 3, 6, and 12 h MDA-MB-231-derived EVs labelled with PKH67 were incubated with MCF7 cells for similar time points. Fluorescent signals indicate the internalization of EVs by cells. Nuclei were counterstained with DAPI (blue). Representative images from three independent experiments are shown.

To understand the patterns of cellular internalization, EVs were labeled with PKH-67 dye and evaluated for their uptake in both autologous and allogenic cells at different time intervals. The uptake was observed through a fluorescence microscope for up to 12 h and quantitatively analyzed by measuring the corrected total cell fluorescence (CTCF) (Figure 3), represent cellular uptake of EVs by BC cells. A progressive increase of PKH-67 fluorescence intensity, starting at 3 h of treatment, confirmed successful uptake of autologous EVs by their parent cells. This suggests that BC cells can recognize and preferentially internalize their own EVs. The uptake was shown to increase till 12 h. The graph of mean CTCF of time-dependent increase in autologous EV uptake by cells was shown in Supplementary Figure S1 **.** In contrast, when EVs originating from 1 cell line were treated to a non-parent BC cell line, no detectable PKH-67 fluorescence was observed at any time point, indicating the absence of EV internalization (Figure 4). The lack of internalization in non-parent cells highlights that BC cells-derived EVs have origin-specific selectivity for internalization. Furthermore, we validated the parent cell-specificity of BC cells-derived EVs using patient-derived HFFs and OSCC cells. The results were consistent with those observed in allogenic cells. There was no visible fluorescence intensity of PKH dye observed until 6 h (Supplementary Figures S2,S3). This confirms that BC cell-derived EVs exhibit specificity for internalization towards parent cells over non-parent cancerous and normal cells. Although these findings indicate preferential internalization of EVs by parent cells, the underlying molecular mechanism remains to be elucidated.

### 3.3 Drug-loading altered physicochemical properties of extracellular vesicles

**FIGURE 5:**
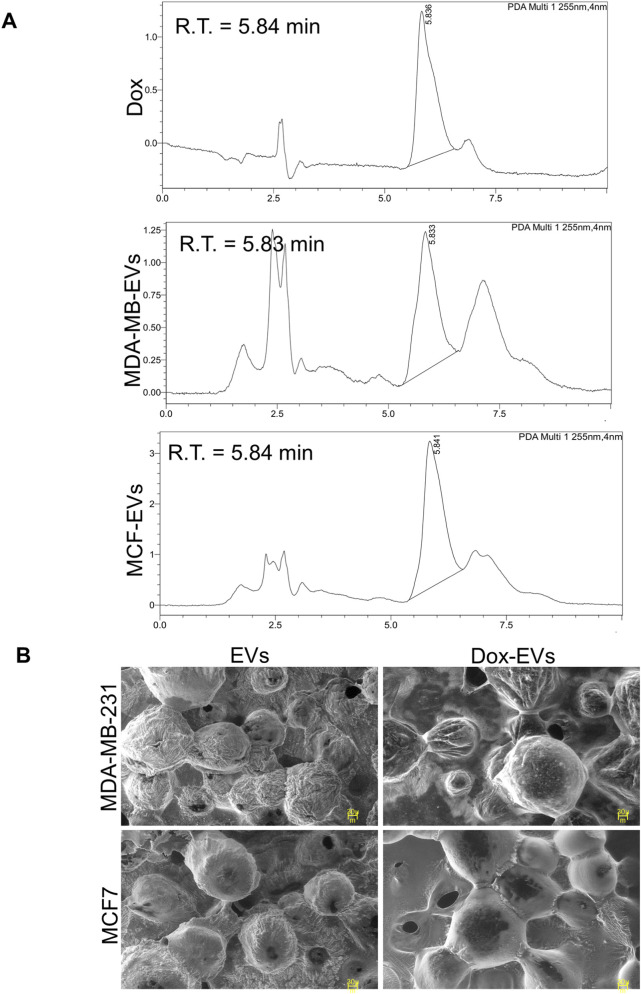
Characterization of doxorubicin (dox) loading in BC cell-derived EVs. **(A)** High-performance liquid chromatography analysis of EVs-dox shows the presence of a retention peak corresponding to dox. **(B)** SEM images showing morphological alterations following dox-loading. Dox-loaded EVs (dox-EVs) appeared slightly enlarged or bulged compared with EVs alone, indicating structural modifications post-drug-loading.

Dox was successfully loaded into BC cell-derived EVs via sonication. After dox-loading, notable changes in entrapment efficiency, average particle size, and concentration were observed in EVs. As shown in Table 1, the particle size of both types of EVs increased to 190.8 ± 71.8 nm for the MDA-MB-231-derived EVs and 210.8 ± 66.3 nm for the MCF7-derived EVs, indicating successful loading of dox. However, the increase in size was accompanied by a reduction in particle count. The entrapment efficiency was 29.8% for MCF7-derived EVs and 22.8% for MDA-MB-231-derived EVs. Furthermore, HPLC analysis confirmed the presence of dox within the EVs. The retention times of dox-EVs closely matched those of free dox, indicating successful drug loading and preservation of its chemical nature and identity (Figure 5A). These findings confirm successful drug loading, potential structural alterations (Figure 5B), and partial loss of vesicles, likely due to the physical stress of sonication and the subsequent purification steps involving differential centrifugation.

**TABLE 1:** Physiological characterization and entrapment efficiency of BC cell-derived EVs after Dox loading.

Parameters	MDA-MB-231		MCF7	
	EVs	Dox-EVs	EVs	Dox-EVs
Particle size (nm)	126.6 ± 58.6	190.8 ± 71.8	163.3 ± 25.7	210.8 ± 66.3
Particle concentration/mL	1.91 x 10^8^	1.09 x 10^8^	1.50 x 10^8^	1.11 x 10^8^
Mean zeta potential (mV)	−0.2	−1.6	−2.5	−1.5
EE (%)	-	22.84	-	29.87
^a^Retention time (t_r_) in min	-	5.83	-	5.84

^a^ Retention time (t_r_), the time from injection to peak detection, was 5.84 min for free doxorubicin. The similar peak in dox-EVs, confirmed the presence of dox in EVs.

### 3.4 Doxorubicin-loaded extracellular vesicles treatment enhances cytotoxicity

**FIGURE 6:**
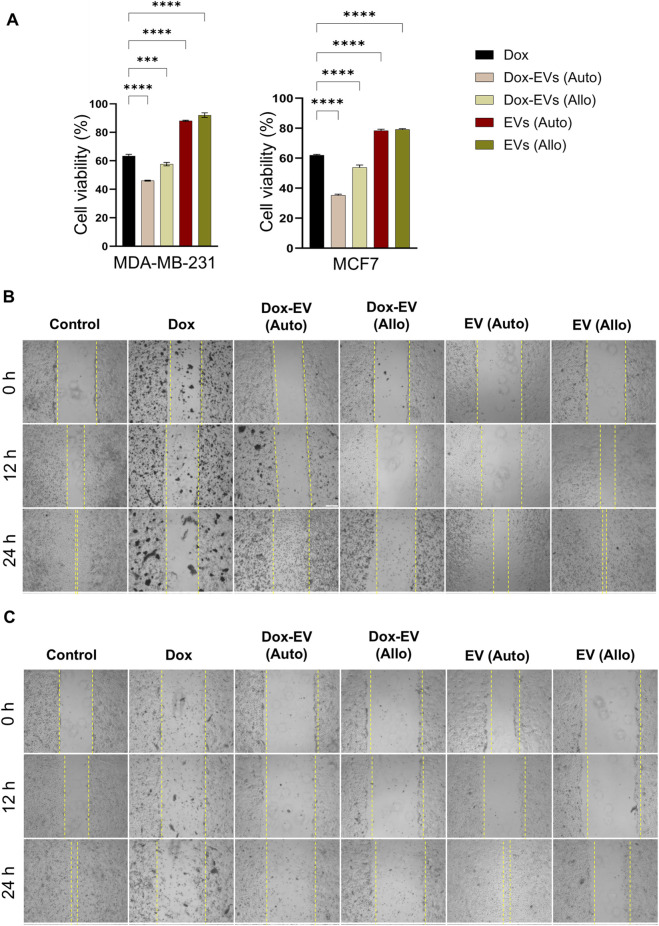
Autologous dox-EVs exhibit enhanced cytotoxicity and anti-migratory effects on parent BC cells. **(A)** MTT assay showing significantly reduced viability of MDA-MB-231 and MCF7 cells treated with autologous dox-EVs compared with free dox, EVs alone, and allogenic dox-EVs. Scratch (wound healing) assays demonstrating the inhibitory effect of dox-EVs on **(B)** MDA-MB-231 cells and **(C)** MCF7 cells. Dox and autologous dox-EV treatments induced extensive cell death, preventing the measurement of wound closure, while allogenic dox-EVs exhibited minimal cell death. The control and EVs alone treatments filled the scratch area. These effects are illustrated by representative images.

The cytotoxicity of dox-EVs, free dox, and EVs alone was assessed using the MTT assay. In MDA-MB-231 cells, free dox reduced cell viability to 63.4% ± 1.2%, whereas autologous dox-EVs further decreased it to 46.1% ± 0.3%, showing enhanced cytotoxicity. However, allogeneic dox-EVs exhibited moderate efficacy (57.7% ± 1.1%), and EVs alone had minimal effect on cell viability (88%–92%), confirming that dox mediated the observed cytotoxicity. Similarly, in MCF-7 cells, treatment of free dox reduced cell viability to 62.1% ± 0.5%, while autologous dox-EVs exhibited the highest effect, reducing cell viability to 35.3% ± 0.7%. Allogeneic dox-EVs induced moderate cytotoxicity (53.9% ± 1.7%), whereas EVs alone had a minimal impact (∼78–79%). Autologous dox-EVs, in both cell lines, consistently demonstrated the highest cytotoxicity, highlighting the importance of parent-cell specificity in EV-mediated drug delivery. EVs alone, irrespective of cell origin, preserved cell viability, indicating that the cytotoxic effects observed in dox-EV treatments were specifically mediated by dox (Figure 6A)**.**

### 3.5 Doxorubicin-loaded extracellular vesicles inhibit wound healing and induce apoptosis

The effects of dox, dox-EVs and EVs alone on BC cell motility and viability were further evaluated using the scratch assay. As shown in Figures 6B,C **,** autologous dox-EVs inhibited healing of the wound and induced extensive cell death, comparable to free fox treatment in both cell lines, demonstrating that dox retained its cytotoxic potential and effectively impaired both viability and healing capacity when delivered via autologous EVs. In contrast, allogeneic dox-EVs caused slight inhibition of wound healing and limited cell death, likely due to lower cellular uptake or reduced compatibility with non-parent cells. However, treatment with EVs alone did not inhibit wound healing but maintained or slightly enhanced cell proliferation, suggesting a pro-survival effect of naive EVs. The quantification of wound healing was not possible for free dox and autologous dox-EV treatments due to extensive cytotoxicity.

**FIGURE 7:**
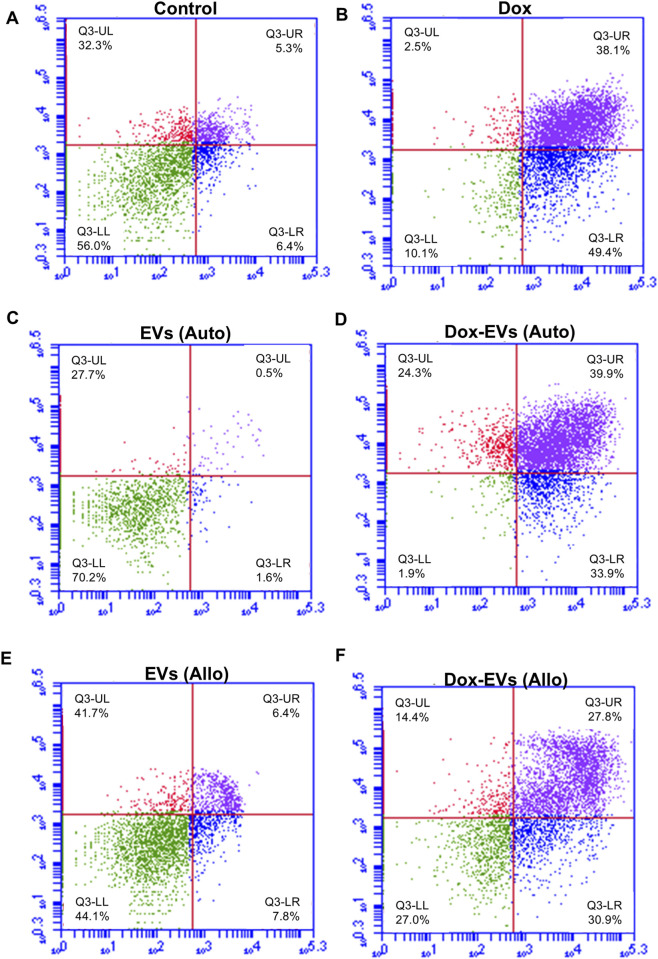
Apoptosis analysis of MDA-MB-231 cells treated with **(A)** Control, **(B)** free dox **(C)** autologous EVs alone **(D)** autologous dox-EVs **(E)** allogenic EVs alone and **(F)** allogenic dox-EVs. The distributions of cells across the live (LL), early apoptotic (LR), late apoptotic (UR), and necrotic (UL) quadrants are presented. Flow cytometry revealed significant apoptosis in cells treated with dox and autologous dox-EVs, with minimal apoptosis observed in cells treated with EVs alone.

**FIGURE 8:**
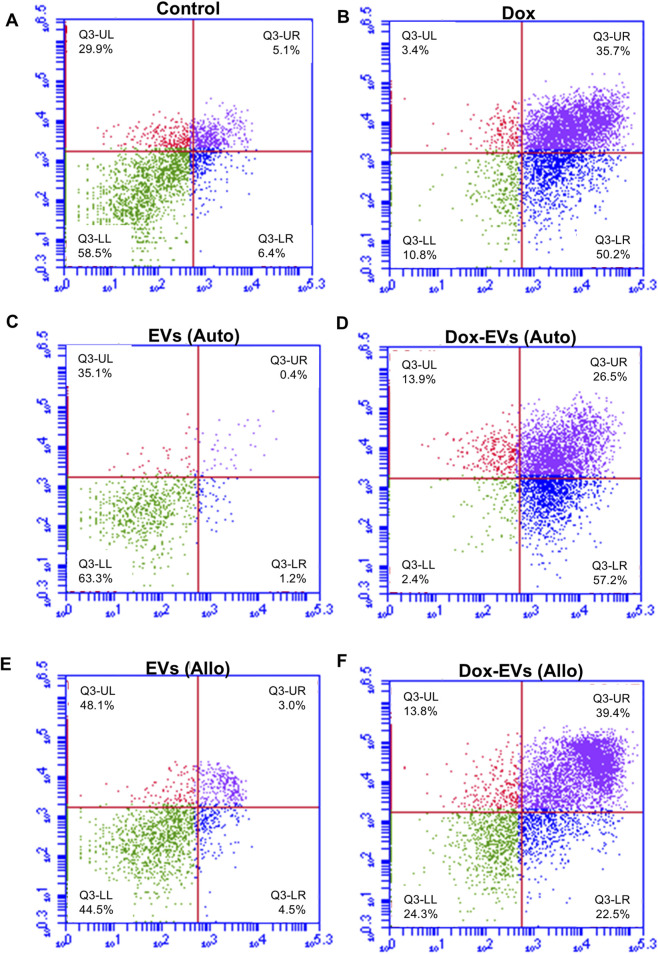
Apoptosis analysis of MCF7 cells treated with **(A)** Control, **(B)** free dox **(C)** autologous EVs alone **(D)** autologous dox-EVs **(E)** allogenic EVs alone and **(F)** allogenic dox-EVs. The distributions of cells across the live (LL), early apoptotic (LR), late apoptotic (UR), and necrotic (UL) quadrants are presented. Flow cytometry revealed significant apoptosis in cells treated with dox and autologous dox-EVs, with minimal apoptosis observed in cells treated with EVs alone.

Further, the apoptotic potential of dox-EVs, free dox and EVs alone was analysed using flow cytometry. The distributions of cells across the live, early apoptotic, late apoptotic, and necrotic quadrants are presented in Figures 7,8 **.** In MDA-MB-231 cells, free dox induced apoptosis in 85.9% of cells (early 50.2%, late 35.7%), while autologous dox-EVs showed a comparable effect (83.7% with early 57.2%, late 26.5%). However, allogeneic dox-EVs induced moderate apoptosis (61.9%; early 22.5%, late 39.4%), whereas EVs alone had minimal effects (1%–7%), similar to those of untreated control cells (11.5%). Similarly, in MCF-7 cells, autologous dox-EVs induced 58.7% apoptosis (early 30.9%, late 27.8%), while free dox induced 87.5% apoptosis (early 49.4%, late 38.1%). Allogeneic dox-EVs induced moderate apoptosis (61.7%; early 33.9%, late 27.8%), whereas EVs alone a had minimal impact (7%–14%), comparable to that of controls (11.7%). Overall, these findings demonstrate that autologous dox-EVs effectively impaired wound healing and induced apoptosis in parent cells compared to free dox or allogeneic dox-EVs, whereas EVs without dox promoted cell survival.

## 4 Discussion

The present study explored the autologous uptake and therapeutic potential of BC cell-derived EVs as nanocarriers for targeted drug delivery. EVs isolated from BC cells were characterized for their physicochemical aspects, investigated for autologous and allogenic uptakes, and evaluated for dox loading and cytotoxic potential. The findings revealed that, though there were notable differences in size, shape and surface charge of the EVs, reflecting the biological properties of parent cells, the uptake patterns and therapeutic efficiency remained consistent. These findings support the potential of BC cells-derived EVs as selective and biocompatible nanocarriers of targeted drug delivery.

The key finding of the present study is preferential and time-dependent uptake of EVs by their parent cell (autologous), with minimal or no observable uptake in allogenic BC cells, OSCC cells and non-cancerous cells. This autologous specificity contrasts with the previous observations, which reported prominent allogenic EV internalization. Several studies have reported the uptake of cancer cells-derived exosomes by allogenic cancer, non-cancerous and bone marrow stromal cells (Feng et al., 2010; Galindo-Hernandez et al., 2013; Verdera et al., 2017; Zheng et al., 2019; Leal-Orta et al., 2022).

However, there are few studies consistent with our findings suggesting autologous uptake patterns of EVs. Wang et., al 2021 also reported significant time-dependent uptake of A549-derived exosomes by autologous cells compared to allogenic cells (Wang et al., 2021), while other studies have reported both energy and time-dependent uptake of exosomes by parent cells (Escrevente et al., 2011; Franzen et al., 2014; Ting HuYan et al., 2018). However, some studies reported both autologous and allogenic internalization of exosomes in pancreatic, lung, and colorectal cancer cells, suggesting that uptake efficiency is greatly influenced by the recipient cell type rather than the donor cell type (Horibe et al., 2018; Kanchanapally et al., 2019). Nevertheless, our findings are consistent with these autologous uptake reports, demonstrating the efficient internalization of EVs by parent BC cells in a time-dependent manner. Notably, no detectable uptake was observed in allogenic BC cells, non-cancerous cells, or OSCC cells. This exclusivity suggests that BC cells can recognize their own EVs through specific molecular signatures, potentially mediated by surface proteins or membrane receptors, such as tetraspanins or integrins, or through specific ligand-receptor interactions, thereby supporting our hypothesis of autologous selectivity. Future proteomic and mechanistic studies are warranted to identify the specific molecular determinants related to this specificity. The lack of detectable allogenic uptake suggests that EVs could minimize off-target accumulation and systemic toxicity which are essential for clinical translation.

After determining the autologous uptake, dox encapsulation and its targeted delivery was assessed. Active loading of dox via sonication induced an increase in EV particle size and a reduction in particle count, indicating structural changes during the drug loading procedure. These findings are consistent with earlier reports that have also described such changes in exosomes post-x-loading (Schindler et al., 2019; Wei et al., 2019). Importantly, along with structural changes, sonication achieved better drug-loading efficiency than previous studies which reported only 12% entrapment of therapeutic drugs in exosomes (Ting HuYan et al., 2018; Wang et al., 2021). The variations in drug loading efficiency between studies may arise from differences in entrapment techniques, EV origins, or the chemical properties of the drugs.

Further, we assessed the therapeutic potential of dox-loaded EVs compared to free dox in both autologous and allogenic cells. Dox-loaded EVs displayed significantly higher cytotoxicity towards autologous BC cells compared to both free dox and EVs alone, suggesting enhanced internalization and drug delivery efficiency. These findings align with those of, Schindler et al., 2019; Wang et al., 2021 who also reported superior cytotoxic effects of drug-loaded exosomes compared to free dox. (Schindler et al., 2019; Wang et al., 2021). The enhanced efficacy likely reflects increased intracellular accumulation of dox mediated by autologous EV internalization. In contrast, EVs alone did not induce cytotoxicity; instead, they promoted cell proliferation, highlighting the intrinsic bioactive nature of cancer cell-derived EVs. Cancer cell-derived EVs were reported as mediators of tumor progression via reprogramming stromal cells and disrupting tissue homeostasis within the tumor microenvironment. The EVs mediate cancer proliferation and progression by transferring their innate cargo, which promotes fibroblast activation, immune evasion, and the formation of a metastatic niche. (Chen et al., 2018; Hinzman et al., 2022; Vulpis et al., 2022). Additionally, the transfer of miRNAs, mRNAs, and proteins has been shown to confer pro-survival functions, proliferative signaling, and therapy resistance in recipient cells, thereby enhancing tumor aggressiveness (Wortzel et al., 2019; LeBleu and Kalluri, 2020). Importantly, the biological effects of EV transfer have been reported to depend on the metastatic state of parent cells (Lázaro-Ibáñez et al., 2017) t. Collectively, these findings highlight the dual role of EVs as both effective therapeutic drug carriers and potential modulators of tumor progression. While EV-mediated drug delivery offers advantages in enhancing the targeted accumulation and efficiency of the drug, the use of EVs alone or unmodified raises important safety concerns due to their inherent oncogenic cargo. Therefore, clinical translations of EV-based therapeutics should avoid administering cancer-derived EVs without characterizing and quantifying their oncogenic contents. Future strategies should focus on cargo depletion or knockdown approaches, along with extensive pre-clinical evaluation of proliferative biomarkers, to mitigate the unwanted pro-tumorigenic effects.

In summary, our findings demonstrate that BC cell-derived EVs exhibit strong autologous specificity, efficient drug loading, and suggest enhanced therapeutic potential, supporting their promise as cell-origin-specific nanocarriers for targeted therapy. The lack of allogenic uptake indicates that EVs could minimize off-target accumulation and effects in healthy tissues, addressing a major challenge posed by current drug delivery systems. However, the proliferative influence of EVs alone emphasizes the need for careful engineering for EV-based therapeutics. While our study provides compelling *in vitro* evidence, it has certain limitations. Firstly, a comprehensive surface proteomic and mechanistic analysis is required for identifying the molecular determinants of autologous recognition and uptake. Second, the use of only TNBC and luminal-type cell lines limits the generalizability of the findings and makes them subtype-specific. Therefore, future studies using a broader spectrum of BC models are warranted. Further, though these *in vitro* findings of autologous specificity and functional advantages are promising, *in vivo* validation is required to evaluate whether they are maintained under physiological conditions. Future investigations for multiple drugs and EV molecular signatures will advance the translational applications of EV-based drug delivery systems. Overall, our results highlight autologous EVs as a promising, selective, and biocompatible platform for precise and personalized drug delivery.

## 5 Conclusion

Our study demonstrates that BC cell-derived EVs can be used for the incorporation of cancer-specific drugs. These drug-loaded EVs serve as efficient nanocarriers for targeted drug delivery and can be effectively implicated for personalized treatment approaches. Although our *in vitro* findings are highly promising, comprehensive *in vivo* validation and in-depth molecular mechanistic studies are warranted to confirm the potential of these findings to support future translational exploration of EV-based drug delivery systems in BC therapeutics.

## Data Availability

The raw data supporting the conclusions of this article will be made available by the authors, without undue reservation.
