# Supplementary figures and images for: Tumor-derived extracellular vesicles mediate cell-specific uptake and facilitate enhanced doxorubicin delivery in breast cancer
Source: Front Pharmacol. 2026 Jan 12;16:1744895. doi: 10.3389/fphar.2025.1744895 (PMC12833375; doi:10.3389/fphar.2025.1744895)

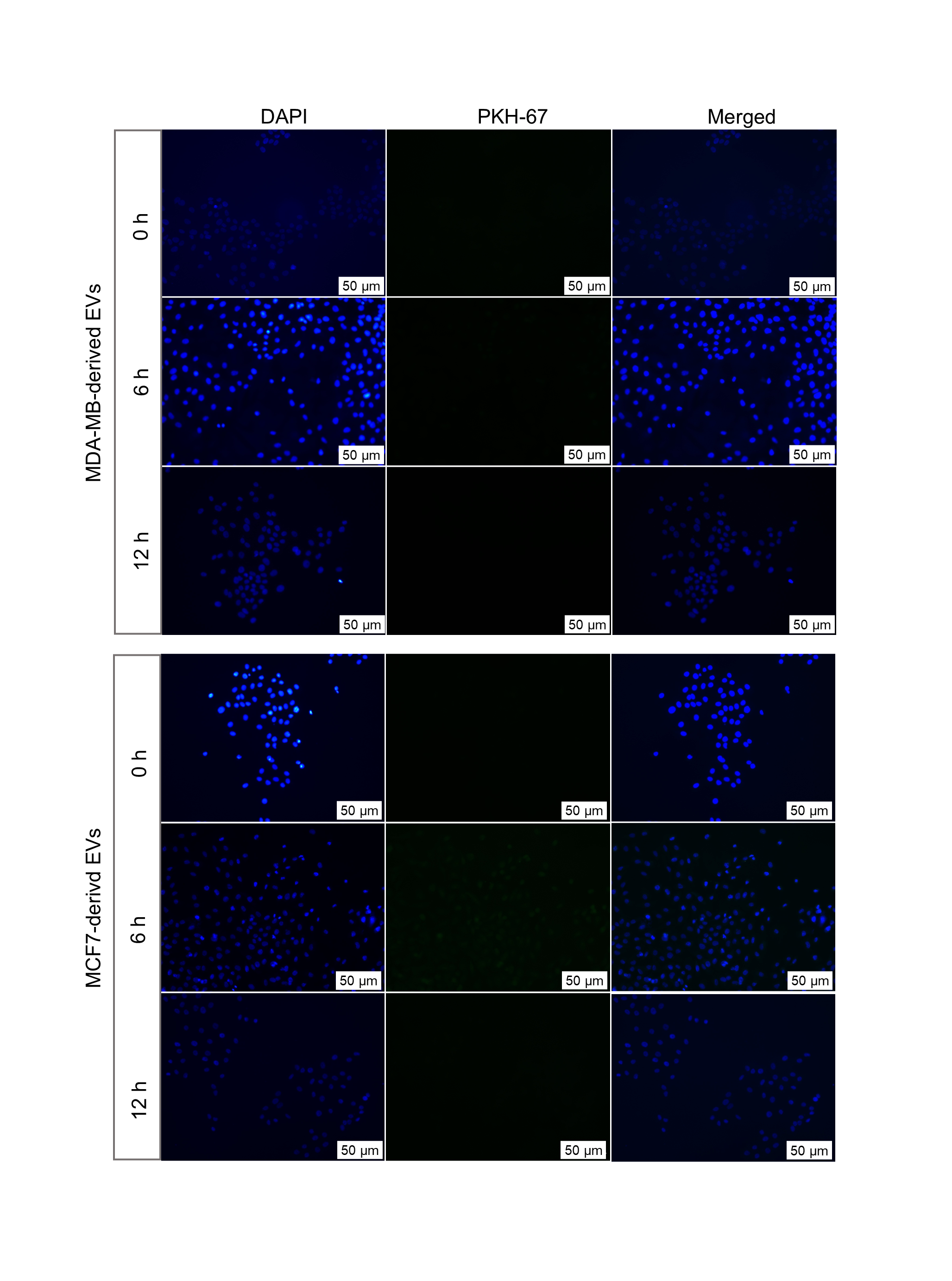

Supplement: Supplementary file 1 [file Image3.tif]

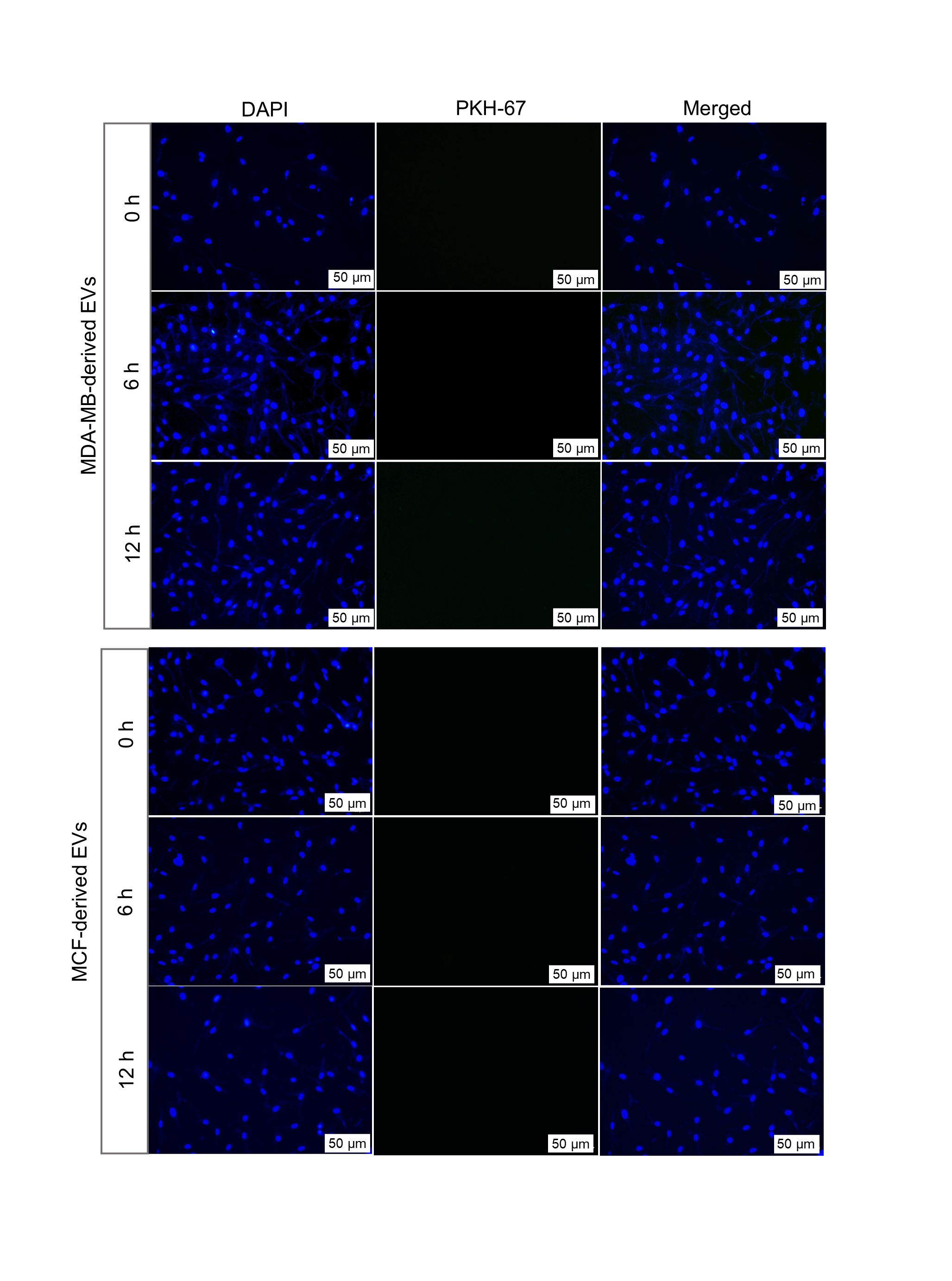

Supplement: Supplementary file 2 [file Image2.tif]

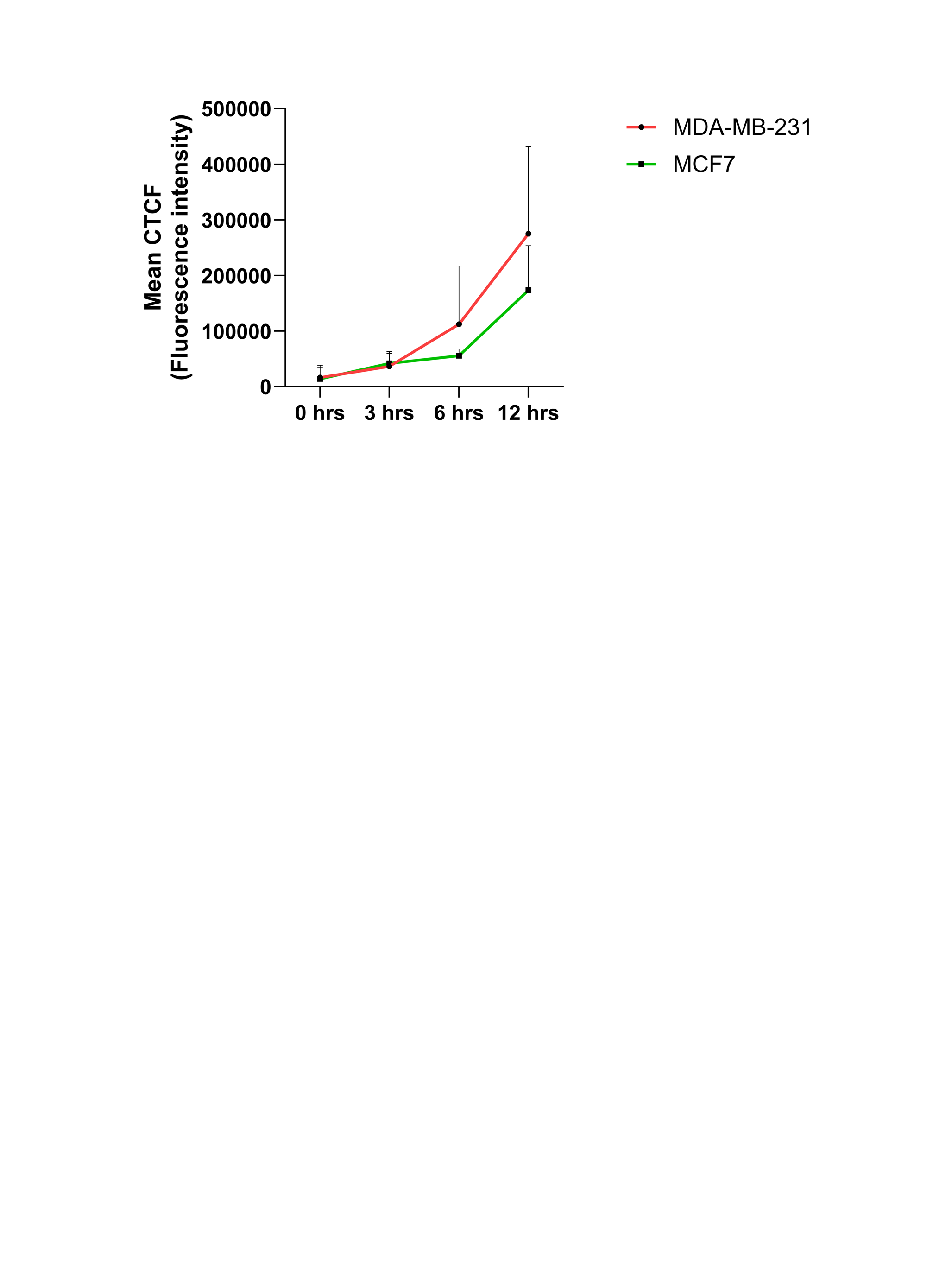

Supplement: Supplementary file 3 [file Image1.tif]
